# LB2302. Ceftobiprole Compared to Daptomycin With or Without Optional Aztreonam for the Treatment of Complicated *Staphylococcus aureus* (SAB): Results of a Phase 3, Randomized, Double-Blind Trial (ERADICATE)

**DOI:** 10.1093/ofid/ofac492.1892

**Published:** 2022-12-15

**Authors:** Thomas L Holland, Sara E Cosgrove, Sarah B Doernberg, Oleksander Pavlov, Ivan Titov, Boyko Atanasov, Maziar Assadi Gehr, Marc Engelhardt, Kamal Hamed, Daniel Ionescu, Mark Jones, Mikael Sauley, Jennifer Smart, Harald Seifert, Timothy C Jenkins, Nicholas A Turner, Vance G Fowler

**Affiliations:** Duke University Medical Center, Durham, North Carolina; Johns Hopkins University Department of Medicine, Baltimore, Maryland; University of California, San Francisco, San Francisco, California; Zaycev V.T. Institute of General and Emergency Surgery of the National Academy of Medical Sciences of Ukraine, Kharkiv, Kharkiv, Kharkivs’ka Oblast', Ukraine; Regional Clinical Hospital, Regional Clinical Hospital, Ivano-Frankivsk Regional Council, Ivano-Frankivsk, Ivano-Frankivs’ka Oblast', Ukraine; Eurohospital Plovdiv, Plovdiv, Plovdiv, Bulgaria; Basilea Pharmaceutica International Ltd, Allschwil, Basel-Landschaft, Switzerland; Basilea Pharmaceutica International Ltd, Allschwil, Basel-Landschaft, Switzerland; Basilea Pharmaceutica International Ltd, Allschwil, Basel-Landschaft, Switzerland; Basilea Pharmaceutica International Ltd, Allschwil, Basel-Landschaft, Switzerland; Basilea Pharmaceutica International Ltd, Allschwil, Basel-Landschaft, Switzerland; Basilea Pharmaceutica International Ltd, Allschwil, Basel-Landschaft, Switzerland; Basilea Pharmaceutica International Ltd, Allschwil, Basel-Landschaft, Switzerland; Institute for Medical Microbiology, Immunology and Hygiene, University of Cologne, Cologne, Nordrhein-Westfalen, Germany; Denver Health and Hospital Authority, Denver, Colorado; Duke University Medical Center, Durham, North Carolina; Duke University Medical Center, Durham, North Carolina

## Abstract

**Background:**

SAB is common, serious, and potentially lethal. Antibiotic options are limited, especially for MRSA. Ceftobiprole is an advanced-generation cephalosporin with bactericidal activity against Gram-positive (including MRSA) and Gram-negative pathogens, with efficacy and safety demonstrated in previous Phase 3 studies in acute bacterial skin infections and pneumonia. The present study evaluated ceftobiprole in patients with complicated SAB.

**Methods:**

ERADICATE was a randomized (1:1), double-blind, multicenter, Phase 3, non-inferiority trial comparing ceftobiprole (BPR) vs daptomycin (DAP) ± optional aztreonam, for up to 42 days of treatment, in patients with complicated SAB (NCT03138733). The primary efficacy endpoint was overall clinical success 70 days post-randomization, adjudicated by a blinded independent Data Review Committee. Success required survival, no new SAB complications, symptom improvement, SAB clearance, and no receipt of other potentially effective antibiotics. The non-inferiority margin for the difference in success rates was -15% (BPR-DAP, 95% CI, 2-sided, lower bound). Safety was assessed through adverse events (AE) and laboratory data.

**Results:**

Of 390 patients randomized, 387 (189 BPR, 198 DAP) were in the modified intent-to-treat (mITT) population who received study medication and had a positive baseline blood culture for *S. aureus* (94 MRSA). Median treatment duration was 21 days for both groups. Key baseline characteristics were balanced (Fig. 1). In the BPR group 69.8% experienced success, compared to 68.7% for DAP (adjusted difference 2.0%, 95% CI -7.1% to 11.1%, Fig. 2). There were no significant differences in mortality, microbiological eradication, or in key subgroup analyses (Fig. 3). The proportion of patients experiencing ≥1 AE was 63% for BPR and 59% for DAP. Treatment-related severe or serious AEs were infrequent. Gastrointestinal AEs, mostly mild nausea, were more frequent with BPR, consistent with data from previous Phase 3 studies.

**Figure:**
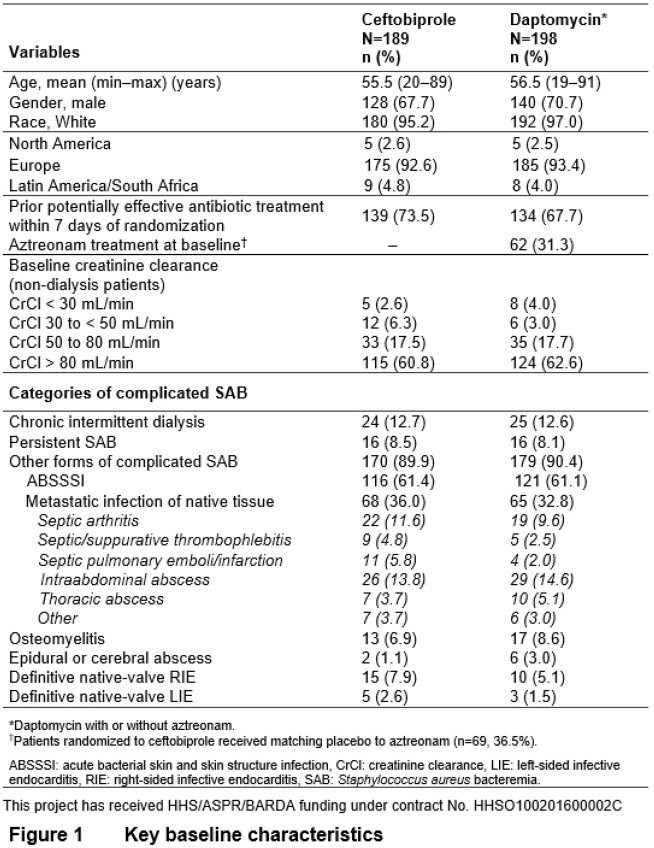

**Figure:**
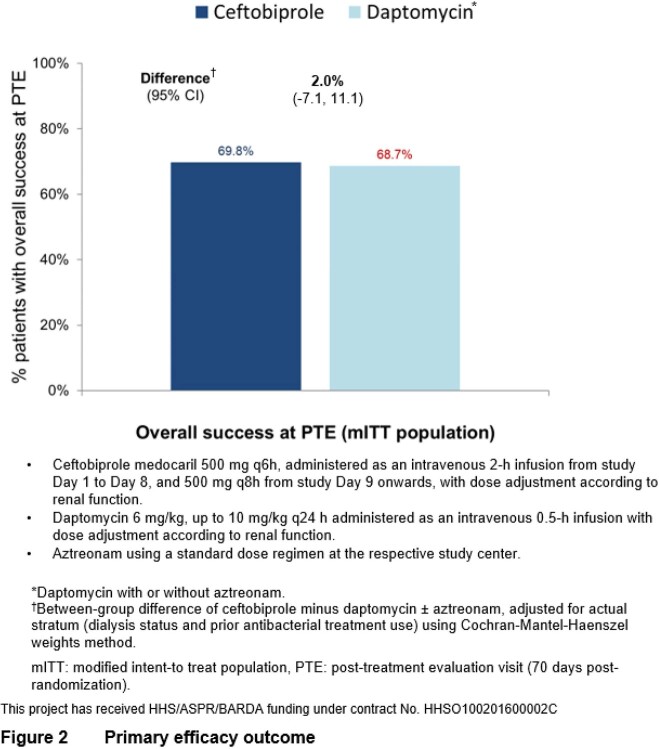

**Figure:**
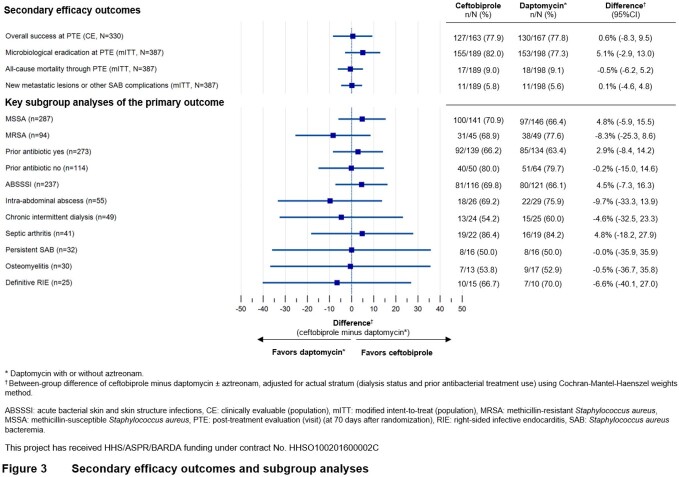

**Conclusion:**

Ceftobiprole is non-inferior to daptomycin for overall success in patients with complicated SAB. All-cause mortality, microbiological eradication rates and new SAB complications were similar between treatment groups. Both treatments were well tolerated.

**Disclosures:**

**Thomas L. Holland, MD**, Aridis: Advisor/Consultant|Basilea Pharmaceutica: Advisor/Consultant|Karius: Advisor/Consultant|Lysovant: Advisor/Consultant **Sara E. Cosgrove, MD**, Basilea: Advisor/Consultant|Debiopharma: Advisor/Consultant **Sarah B. Doernberg, MD, MAS**, Basilea: Advisor/Consultant|Genentech: Advisor/Consultant|Gilead: Grant/Research Support|Johnson and Johnson: Advisor/Consultant|NIH: Grant/Research Support|Regeneron: Grant/Research Support **Maziar Assadi Gehr, MD**, Basilea Pharmaceutica: full time employee of Basilea Pharmaceutica International Ltd **Marc Engelhardt, MD**, Basilea Pharmaceutica: full time employee of Basilea Pharmaceutica International Ltd **Kamal Hamed, MD**, Basilea Pharmaceutica: previous full time employee of Basilea Pharmaceutica International Ltd|Lysovant: full time employee of Lysovant **Daniel Ionescu, MD**, Basilea Pharmaceutica: full time employee of Basilea Pharmaceutica International Ltd **Mark Jones, PhD**, Basilea Pharmaceutica: full time employee of Basilea Pharmaceutica International Ltd **Mikael Sauley, MSc**, Basilea Pharmaceutica: full time employee of Basilea Pharmaceutica International Ltd **Jennifer Smart, PhD**, Basilea Pharmaceutica: full time employee of Basilea Pharmaceutica International Ltd **Harald Seifert, MD**, Basilea Pharmaceutica: Advisor/Consultant|Debiopharm: Advisor/Consultant|Eumedica: Advisor/Consultant|Gilead: Advisor/Consultant|MSD: Advisor/Consultant|Shionogi: Advisor/Consultant **Timothy C. Jenkins, MD**, Basilea: Clinical outcomes adjudication committee **Vance G. Fowler, Jr, MD, MHS**, Armata Valanbio Akagera Aridis Roche: Advisor/Consultant|BASILEA: Grant/Research Support|Basilea Novartis Debiopharm Genentech: Advisor/Consultant|MedImmune Bayer Janssen Contrafect Regeneron Destiny Amphliphi Integrated Bioth: Advisor/Consultant|NIH MedImmune Allergan Theravance Novartis Merck Contrafect Karius Genentech Regeneron Janssen: Grant/Research Support.

